# Association of Childhood Oral Infections With Cardiovascular Risk Factors and Subclinical Atherosclerosis in Adulthood

**DOI:** 10.1001/jamanetworkopen.2019.2523

**Published:** 2019-04-26

**Authors:** Pirkko J. Pussinen, Susanna Paju, Jaana Koponen, Jorma S. A. Viikari, Leena Taittonen, Tomi Laitinen, David P. Burgner, Mika Kähönen, Nina Hutri-Kähönen, Olli T. Raitakari, Markus Juonala

**Affiliations:** 1 Oral and Maxillofacial Diseases, University of Helsinki and Helsinki University Hospital, Helsinki, Finland; 2 Department of Medicine, University of Turku, Turku, Finland; 3 Division of Medicine, Turku University Hospital, Turku, Finland; 4 Vaasa Central Hospital, Vaasa, Finland; 5 Department of Clinical Physiology, University of Eastern Finland and Kuopio University Hospital, Kuopio, Finland; 6 Royal Children’s Hospital, Parkville, Victoria, Australia; 7 Murdoch Childrens Research Institute, Parkville, Victoria, Australia; 8 Department of Clinical Physiology, Tampere University Hospital and University of Tampere, Tampere, Finland; 9 Department of Pediatrics, University of Tampere and Tampere University Hospital, Tampere, Finland; 10 Research Center of Applied and Preventive Cardiovascular Medicine, University of Turku, Turku, Finland; 11 Department of Clinical Physiology and Nuclear Medicine, Turku University Hospital, Turku, Finland

## Abstract

**Importance:**

Severe forms of common chronic oral infections or inflammations are associated with increased cardiovascular risk in adults. To date, the role of childhood oral infections in cardiovascular risk is not known because no long-term studies have been conducted.

**Objective:**

To investigate whether signs of oral infections in childhood are associated with cardiovascular risk factors and subclinical atherosclerosis in adulthood.

**Design, Setting, and Participants:**

The cohort study (n = 755) was derived from the Cardiovascular Risk in Young Finns Study, an ongoing prospective cohort study in Finland initiated in 1980. Participants underwent clinical oral examinations during childhood, when they were aged 6, 9, or 12 years and a clinical cardiovascular follow-up in adulthood in 2001 at age 27, 30, or 33 years and/or in 2007 at age 33, 36, or 39 years. Cardiovascular risk factors were measured at baseline and during the follow-up until the end of 2007. Final statistical analyses were completed on February 19, 2019.

**Main Outcomes and Measures:**

Four signs of oral infections (bleeding on probing, periodontal probing pocket depth, caries, and dental fillings) were documented. Cumulative lifetime exposure to 6 cardiovascular risk factors was calculated from dichotomized variables obtained by using the area-under-the-curve method. Subclinical atherosclerosis (ie, carotid artery intima-media thickness [IMT]) was quantified in 2001 (n = 468) and 2007 (n = 489).

**Results:**

This study included 755 participants, of whom 371 (49.1%) were male; the mean (SD) age at baseline examination was 8.07 (2.00) years. In this cohort, 33 children (4.5%) had no sign of oral infections, whereas 41 (5.6%) had 1 sign, 127 (17.4%) had 2 signs, 278 (38.3%) had 3 signs, and 248 (34.1%) had 4 signs. The cumulative exposure to risk factors increased with the increasing number of oral infections both in childhood and adulthood. In multiple linear regression models, childhood oral infections, including signs of either periodontal disease (*R*^2^ = 0.018; *P* = .01), caries (*R*^2^ = 0.022; *P* = .008), or both (*R*^2^ = 0.024; *P* = .004), were associated with adulthood IMT. The presence of any sign of oral infection in childhood was associated with increased IMT (third tertile vs tertiles 1 and 2) with a relative risk of 1.87 (95% CI, 1.25-2.79), whereas the presence of all 4 signs produced a relative risk of 1.95 (95% CI, 1.28-3.00). The associations were more obvious in boys: if periodontal disease were present, the corresponding estimate was 1.69 (95% CI, 1.21-2.36); if caries, 1.46 (95% CI, 1.04-2.05); and if all 4 signs of oral infections, 2.25 (95% CI, 1.30-3.89). The associations were independent of cardiovascular risk factors.

**Conclusions and Relevance:**

Oral infections in childhood appear to be associated with the subclinical carotid atherosclerosis seen in adulthood.

## Key Points

**Question:**

Are childhood oral infections or inflammatory conditions associated with the risk of adulthood subclinical atherosclerosis?

**Findings:**

In this cohort study of 755 participants followed up for 27 years into adulthood, a number of clinical signs of oral infections in childhood were associated with both cumulative exposure to cardiovascular risk factors during the follow-up and subclinical atherosclerosis in adulthood.

**Meaning:**

Childhood oral infections may be a modifiable risk factor for adult cardiovascular disease.

## Teaser

This cohort study examines signs of oral infection in children and the risk factors for atherosclerosis that develop as the children mature to adulthood, in this ongoing follow-up study in Finland.

## Introduction

Oral infections (periodontal diseases and dental caries) are among the most common infection-induced inflammatory diseases worldwide. In Finland, the prevalence in adults of periodontal diseases is 75% and caries or fillings is 79%.^1,2^ Both diseases initiate early in life: caries and gingivitis are the most common chronic diseases, with an incidence of 59% for caries and 80% for gingivitis in American adolescents.^3^ If early signs go untreated, these diseases may lead to more severe periodontal and endodontal infections, both of which may result in tooth loss.

The role of periodontitis as an independent risk factor for atherosclerotic cardiovascular diseases (CVDs) is established, although the evidence does not support causality.^4^ The oral dysbiosis and the local inflammation in periodontitis contribute to systemic inflammatory burden by dispersing inflammatory mediators and bacterial stimuli in the circulation.^5^ Periodontal treatment improves the atherosclerotic profile by reversing endothelial dysfunction and reducing inflammatory and lipid biomarkers.^6^ Given that endodontic infections share several characteristics with periodontitis, including altered microbiota and proinflammatory mediators, their role in CVD is investigated.^7^

Early life exposure to cardiovascular risk factors, such as high blood pressure, increased body mass index (BMI), the proatherogenic lipid profile, and smoking, is associated with the development of atherosclerosis in adulthood.^8^ Identifying all childhood risk factors is important because improvements in risk factor burden by young adulthood are advantageous for cardiometabolic health in middle age.^9^ The role of childhood oral infections in cardiovascular risk is poorly understood, and to date no long-term studies have been conducted, to our knowledge. In participants aged 12 to 19 years, subgingival microorganism clusters were not associated with CVD risk factors,^10^ whereas dental caries in adolescents may be associated with obesity and an unfavorable risk profile.^11,12^ Thus, we investigated the association of childhood oral infections with preclinical carotid atherosclerosis in a follow-up study of 27 years. In addition, we examined the association between the number of signs of oral infections in childhood and cardiovascular risk factor profiles during the follow-up.

## Methods

This study complies with the Declaration of Helsinki,^13^ and the Ethics Committee of the Hospital District of Southwest Finland approved the research protocol. Written informed consent was obtained from the participants or their parents. This study followed the Strengthening the Reporting of Observational Studies in Epidemiology (STROBE) reporting guideline.

### Population

We obtained participants from the ongoing Cardiovascular Risk in Young Finns Study, the details of which, including analyses of attrition to show the representativeness of the cohort, have been published previously.^14^ This present population included 755 participants who underwent a baseline evaluation (including a dental examination) in 1980 at age 6, 9, or 12 years and a clinical cardiovascular follow-up in adulthood in 2001 at age 27, 30, or 33 years and/or in 2007 at age 33, 36, or 39 years. In the present analyses, the follow-up period lasted until the end of 2007. This population represented a random subpopulation of these age groups of the whole cohort. Final statistical analyses were completed on February 19, 2019.

### Oral Examinations

Oral examinations were performed in 1980, when the 755 participants were children aged 6, 9, or 12 years. Children were examined at university dental schools in 5 major cities (Helsinki, Kuopio, Tampere, Turku, and Oulu) in Finland. Oral hygiene habits in the form of brushing frequency per day was obtained from a questionnaire completed by the participants or their parents. The oral examination recorded the number of teeth (both deciduous and permanent) and measured present or previous (treated) dental infections (caries and fillings) and periodontal diseases (gingival bleeding on probing and periodontal probing pocket depths). The presence of caries and fillings were recorded from 5 surfaces (mesial, buccal, distal, lingual, and occlusal) of both permanent and deciduous teeth. Periodontal probing was performed on 2 sites (upper teeth: mesial and buccal; lower teeth: mesial and lingual) of 6 index teeth (upper teeth: right first molar, left central incisor, and left first premolar; lower teeth: left first molar, right central incisor, and right first premolar). Probing pocket depths of the gingival sulcus were categorized as no pocketing (0-1.9 mm), slight gingival deepening and shallow periodontal pockets (2-5.9 mm), and deep periodontal pockets (≥6 mm). Bleeding on probing was observed after probing and recorded as present or absent.

### Cardiovascular Risk Factors

Height (rounded to the nearest 1 cm) and weight (rounded to the nearest 0.1 kg) were measured at all time points using a similar protocol, and BMI was calculated (weight in kilograms divided by height in meters squared). Baseline blood pressure was measured by a mercury sphygmomanometer, and during follow-up, a random 0-mercury sphygmomanometer was used. Blood samples were obtained after a 12-hour fast. Standard enzymatic methods were used to obtain levels of serum total cholesterol, triglycerides, low-density lipoprotein and high-density lipoprotein (HDL) cholesterol, C-reactive protein, and plasma glucose. Cholesterol, triglyceride, and glucose concentrations are presented as milligrams per deciliter after using molecular weights of 180 g/mol for cholesterol, 387 g/mol for triglycerides, and 875 g/mol for glucose. (To convert milligrams per deciliter to millimoles per liter, multiply by 0.0259 for cholesterol; by 0.0113 for triglycerides; and by 0.0555 for glucose.) The questionnaire requested family income information with an 8-category scale (1 indicating low, and 8 high).

### Carotid Artery Intima-Media Thickness

Ultrasonographic studies were performed using ultrasound mainframes (Sequoia 512; Acuson) with 13.0 MHz linear array transducers.^8^ A similar scanning protocol was used in 2001 and 2007. Intima-media thickness (IMT) was measured on the posterior (far) wall of the left carotid artery. At least 4 measurements were taken approximately equal to 10 mm proximal to the bifurcation to obtain the mean carotid artery IMT. The digitally stored scans were manually analyzed by 1 reader (M.J., the same reader for both the 2001 and 2007 follow-up) who was blinded to participants’ details. To assess the intraindividual reproducibility of ultrasonographic measurements, 57 participants were reexamined 3 months after the initial visit. The between-visit coefficient of variation was 6.4%.^8^ The number of participants examined were 468 in 2001 and 489 in 2007.

### Statistical Analysis

The significance of the differences between continuous and categorical variables was analyzed with the unpaired 2-tailed *t* test, analysis of variance, Mann-Whitney, or χ^2^ test, when appropriate. In all analyses, a 2-sided *P* < .05 was considered statistically significant. In the tables, we used means and SDs to describe the data, and in the figures, we used means and SEs for clarity. Cardiovascular risk factors, including BMI, systolic and diastolic blood pressure, and plasma HDL and low-density lipoprotein cholesterol, triglycerides, and glucose concentrations measured during the follow-up were dichotomized through the area-under-the-curve method.^15^ First, we performed receiver operating characteristic curve analyses for all risk factors. Each risk factor was the test variable, and the mean IMT-2007 was the state variable. Then, we obtained the cutoff values from the area-under-the-curve coordinate corresponding to a specificity of 0.75 (eTable 1 in the Supplement). Values above the cutoff level were recoded as 1 and values below the level as 0. For HDL cholesterol concentration, the values were recorded the other way around. Cumulative exposure to the risk factors was calculated by summing the dichotomized values separately for childhood (1980, 1983, and 1986), adulthood (2001 and 2007), and the whole follow-up time.

Associations between the mean IMT as a continuous variable and signs of oral infections were analyzed by multiple linear regression models, adjusted for the cumulative exposure to the risk factors. The sum of standardized values for signs of caries (caries and fillings) was used because of the skewness of the oral variables. The associations between signs of oral infections and the IMT (tertiles 1 and 2 vs tertile 3) were analyzed in a Poisson regression model by using the oral variables as continuous and categorized (present vs absent) variables. In the models, in which the oral variables were used as continuous variables, they were first standardized and then summed up. The models were adjusted for age and sex first and then further adjusted for the cumulative exposure to the risk factors, smoking (yes or no), and family income. The regression models were controlled for possible selection bias owing to missing data by using the inverse probability weighting. The unmeasured confounding owing to the observational study design was estimated by the E value.^16^

## Results

The characteristics of the study population of 755 (371 [49.1%] were male; mean [SD] age at baseline examination of 8.07 [2.00] years) and the risk factors in 1980 are shown in Table 1. Children with signs of periodontal disease, compared with the children without such signs, were older (mean [SD] age, 8.26 [1.89] years vs 7.56 [2.22] years) and had higher BMI (mean [SD] BMI, 16.9 [2.3] vs 16.5 [2.1]) and diastolic blood pressure (mean [SD] diastolic blood pressure, 68.4 [9.3] vs 65.5 [8.8] mm Hg). Children with treated or untreated caries, compared with children without these signs, were older (mean [SD] age, 8.26 [1.92] vs 6.46 [1.89] years), had higher BMI (mean [SD] BMI, 16.9 [2.3] vs 15.7 [1.8]), systolic blood pressure (mean [SD] systolic blood pressure, 111 [9.6] vs 106 [9.6] mm Hg), diastolic blood pressure (mean [SD] diastolic blood pressure, 68.1 [9.4] vs 65.6 [8.1] mm Hg), lower family income (mean [SD] income category, 5.22 [1.76] vs 5.69 [1.85]), and lower C-reactive protein levels (median [interquartile range] C-reactive protein, 0.20 [0.90] vs 0.29 [0.90] mg/L). The baseline characteristics between those with (n = 489) and those without (n = 266) the IMT measurements in 2007 differed only in sex; girls were more often examined than boys (57.5% vs 44.6%) (eTable 2 in the Supplement).

**Table 1.** Characteristics of the Study Population at Baseline in 1980

Variable	All Participants (N = 755)	Signs of Periodontal Disease^a^		*P* Value	Signs of Caries^b^		*P* Value
		No (N = 129)	Yes (N = 597)		No (N = 99)	Yes (N = 656)	
Male, No. (%)^c^	371 (49.1)	57 (44.2)	297 (49.7)	.25	49 (49.5)	322 (50.9)	.94
Age, mean (SD), y^d^	8.07 (2.00)	7.56 (2.22)	8.26 (1.89)	<.001	6.46 (1.89)	8.26 (1.92)	<.001
BMI, mean (SD), ^d^	16.8 (2.3)	16.5 (2.1)	16.9 (2.3)	.04	15.7 (1.8)	16.9 (2.3)	<.001
Systolic blood pressure, mean (SD), mm Hg^d^	110 (9.7)	111 (10.2)	110 (9.6)	.46	106 (9.6)	111 (9.6)	<.001
Diastolic blood pressure, mean (SD), mm Hg^d^	67.8 (9.2)	65.5 (8.8)	68.4 (9.3)	.001	65.6 (8.1)	68.1 (9.4)	.01
Total cholesterol, mean (SD), mg/dL ^d^	209.2 (34.0)	211.5 (35.6)	206.5 (34.0)	.14	205.0 (34.4)	208.0 (34.0)	.40
LDL cholesterol, mean (SD), mg/dL^d^	133.8 (30.9)	138.1 (32.5)	132.6 (30.9)	.08	133.4 (30.9)	134.2 (31.3)	.83
HDL cholesterol, mean (SD), mg/dL^d^	63.0 (12.0)	62.6 (11.6)	63.0 (12.0)	.69	61.5 (12.0)	63.4 (12.0)	.12
Triglycerides, mean (SD), mg/dL^d^	53.1 (24.8)	54.0 (30.1)	54.0 (23.9)	.94	51.4 (19.5)	54.0 (24.8)	.41
Family income class, mean (SD)^e^	5.27 (1.78)	5.46 (1.81)	5.24 (1.77)	.21	5.69 (1.85)	5.22 (1.76)	.01
CRP, median (IQR), mg/L^f^	0.21 (0.44)	0.22 (0.60)	0.20 (0.42)	.51	0.29 (0.90)	0.20 (0.39)	.02

Abbreviations: BMI, body mass index (calculated as weight in kilograms divided by height in meters squared); CRP, C-reactive protein; HDL, high-density lipoprotein; IQR, interquartile range; LDL, low-density lipoprotein.

SI conversion factors: To convert total, LDL, and HDL cholesterol to millimoles per liter, multiply by 0.0259; triglycerides to millimoles per liter, multiply by 0.0113; and C-reactive protein to nanomoles per liter, multiply by 9.524.

^a^ Bleeding on probing or increased probing pocket depths.

^b^ Caries or fillings.

^c^ χ^2^ Test.

^d^ Unpaired 2-tailed *t* test.

^e^ Family income is based on an 8-category scale (1 indicating low, and 8 high).

^f^ Mann-Whitney test (n = 486).

### Signs of Oral Infections

The mean values, percentages, and distributions of the signs of oral infections (ie, number of surfaces with caries, number of teeth with fillings, and percentage of sites with gingival bleeding and increased probing pocket depths) are shown in eTable 2 and the eFigure in the Supplement. Bleeding was detected in 511 participants (67.7%), caries in 656 (86.9%), and fillings in 621 (82.3%), with no sex differences. Slightly increased pocket depth was found in 391 participants (53.9%), and it was a more prevalent finding in boys than girls (59.9% vs 48.9%; *P* = .006). Deep periodontal pockets were not found. The distribution of participants in the categories of signs of oral infections was as follows: 33 children (4.5%) had no sign of oral infections, whereas 41 (5.6%) had 1 sign, 127 (17.4%) had 2 signs, 278 (38.3%) had 3 signs, and 248 (34.1%) had 4 signs, and the mean number of signs did not differ between boys and girls (2.97 [1.05] vs 2.87 [1.08]; *P* = .23). Most children (688 [91.2%]) were reported to brush their teeth daily, but a percentage of the boys and the girls (12.2% vs 5.6%; *P* < .001) were not.

### Atherosclerosis Risk Factors in Oral Infections

**Figure 1.**
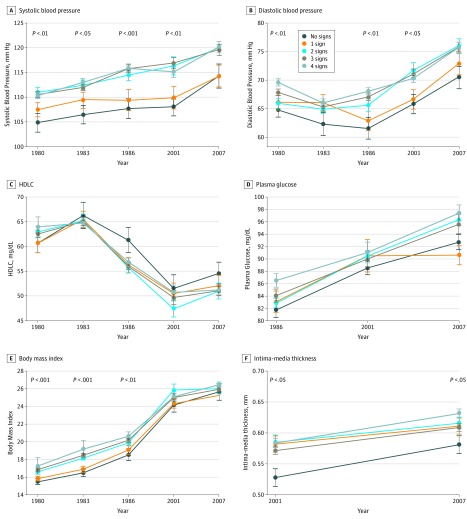
Atherosclerosis Risk Factors and Carotid Artery Intima-Media Thickness According to Number of Clinical Signs of Oral Infections The numbers of observations in different years are as follows: A, systolic blood pressure (n = 727 in 1980, 639 in 1983, 563 in 1986, 470 in 2001, 472 in 2007); B, diastolic blood pressure (n = 726 in 1980, 634 in 1983, 563 in 1986, 470 in 2001, 472 in 2007); C, high-density lipoprotein cholesterol (HDLC) concentration (n = 725 in 1980, 626 in 1983, 559 in 1986, 474 in 2001, 475 in 2007); D, plasma glucose concentration (n = 548 in 1986, 474 in 2001, 476 in 2007); E, bod wheny mass index (n = 727 in 1980, 638 in 1983, 563 in 1986, 469 in 2001, 470 in 2007); and F, intima-media thickness (n = 468 in 2001, 489 in 2007). Mean (SE [error bars]) values and the statistical difference between the groups are shown.

The atherosclerosis risk factors across the follow-up period and at the time of the 2 IMT measurements are presented according to the number of signs of oral infections (Figure 1). Both systolic and diastolic blood pressure values as well as BMI differed statistically significantly between the groups: participants with no sign of oral infections had the lowest values throughout the follow-up. The largest mean (SD) differences between those with no sign and those with 4 signs of oral infections were observed in 1986 for systolic blood pressure (8.1 [2.4] mm Hg; *P* = .001) and diastolic blood pressure (6.5 [1.9] mm Hg; *P* = .001) and in 1983 for BMI (2.9 [0.5]; *P* < .001). Similar but nonsignificant trends were seen in HDL cholesterol and plasma glucose concentrations: Those with no sign of oral infections mainly had the highest HDL cholesterol and lowest glucose concentrations. The mean (SD) differences were highest in 1986 for both HDL cholesterol (4.5 [2.2] mg/dL; *P* = .04) and plasma glucose (4.7 [2.9] mg/dL; *P* = .11) concentrations. Serum low-density lipoprotein cholesterol and triglyceride concentrations did not differ statistically significantly between the groups.

**Figure 2.**
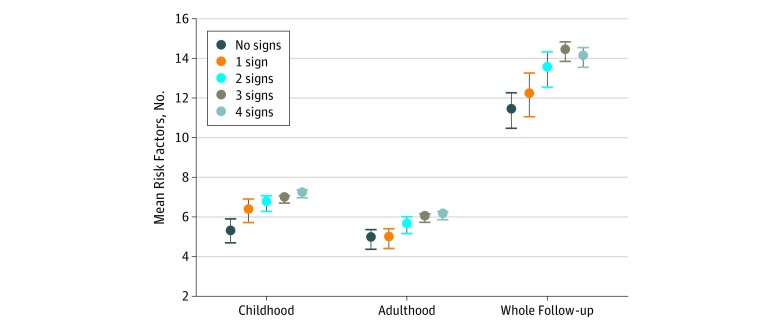
Cumulative Exposure to Atherosclerosis Risk Factors According to Number of Clinical Signs of Oral Infections The risk factors are systolic and diastolic blood pressure; body mass index; and concentrations of glucose, triglycerides, high-density lipoprotein cholesterol, and low-desnity lipoportein cholesterol for childhood in 1980, 1983, and 1986, and for adulthood in 2001 and 2007. The area-under-the-curve variables for risk factors were dichotomized 1×1 into high-risk (≥75th percentile) and low-risk (<75th percentile) factor levels and then summed. Mean (SE [error bars]) values and statistically significant linear terms are shown.

Cumulative exposure to the risk factors, including blood pressure values and BMI as well as plasma lipid and glucose concentrations measured during the 5 appointments, is presented for childhood, adulthood, and the whole follow-up time (Figure 2). The mean (SE) number of risk factors increased with the increasing signs of oral infections in adulthood (mean [SE] for no sign, 4.9 [0.5]; for 1 sign, 4.91 [0.5]; for 2 signs, 5.6 [0.4]; for 3 signs, 5.98 [0.2]; for 4 signs, 6.1 [0.2]; *P* for linear trend = 0.04) and during the whole follow-up (mean [SE] for no sign, 11.4 [0.9]; for 1 sign, 12.2 [1.1]; for 2 signs, 13.5 [0.9]; for 3 signs, 14.4 [0.5]; for 4 signs, 14.1 [0.5]; *P* = .01), but it was especially obvious in childhood (mean [SE] for no sign, 5.31 [0.6]; for 1 sign, 6.34 [0.6]; for 2 signs, 6.71 [0.4]; for 3 signs, 6.91 [0.2]; for 4 signs, 7.2 [0.2]; *P* = .008) when the clinical oral examinations were performed.

### Subclinical Atherosclerosis and Signs of Oral Infections

The IMT measurements in 2001 and 2007 follow-up differed statistically significantly between the groups on the basis of oral infections, with the lowest values in those with no sign of oral infections (Figure 1F). The mean (SD) difference in IMT measurement between those with no sign and those with 4 signs was 0.056 (0.019) mm (*P* = .004) in 2001 and 0.051 (0.017) mm (*P* = .003) in 2007. In a linear regression model adjusted for the cumulative exposure to the risk factors, caries and fillings (β, 0.145; *P* = .008), bleeding and pocketing (β, 0.135; *P* = .01), and number of signs of oral infections (β, 0.158; *P* = .004) were associated directly with the IMT in 2007 (Table 2). In multiple linear regression models, childhood oral infections, including signs of either periodontal disease (*R*^2^ = 0.018; *P* = .01), caries (*R*^2^ = 0.022; *P* = .008), or both (*R*^2^ = 0.024; *P* = .004), were associated with adulthood IMT (Table 2).

**Table 2.** Linear Regression Analysis of the Association Between Carotid Artery Intima-Media Thickness and Signs of Oral Infections

Independent Variable	Dependent Variable: Mean IMT in 2007^a^		
	Standardized β	*P* Value	*R*^2^
Signs of periodontal disease^b^	0.137	.01	0.018
Signs of caries^c^	0.145	.008	0.022
Signs of oral infections^d^	0.158	.004	0.024

Abbreviation: IMT, intima-media thickness.

^a^ Multiple linear regression adjusted for the cumulative exposure to cardiovascular disease risk factors during follow-up classified with the area-under-the-curve method. The models were weighed by using inverse probability score. *R*^2^ values are reported from corresponding unadjusted simple linear regressions with fitting models.

^b^ Number of sites with signs of periodontal disease (bleeding on probing and increased probing pocket depth).

^c^ Sum of standardized values for number of surfaces with caries and teeth with fillings.

^d^ Number of signs of oral infections (bleeding on probing, increased probing pocket depth, caries, and fillings).

The presence of any sign of oral infection in childhood was associated with increased IMT (third tertile vs tertiles 1 and 2) with a relative risk of 1.87 (95% CI, 1.25-2.79), whereas the presence of all 4 signs produced a relative risk of 1.95 (95% CI, 1.28-3.00). In a Poisson regression, the dependent variable was the IMT tertile (third tertile vs tertiles 1 and 2). When analyzed as continuous variables, the sum of all oral infections was associated with the highest IMT tertile (risk ratio [RR], 1.031; 95% CI, 1.000-1.064) (eTable 3 in the Supplement). When analyzed as categorical variables, the presence of either active or inactive caries was associated with the IMT (RR, 1.42; 95% CI, 1.07-1.88). Similarly, 1 to 3 signs of oral infections were associated with the IMT (RR, 1.74; 95% CI, 1.16-2.62) and so were 4 signs (RR, 1.95; 95% CI, 1.28-3.00). The risk was especially clear in boys: when all 4 signs were present, they had an RR of 2.25 (95% CI, 1.30-3.89), for the highest IMT tertile 27 years later (Table 3). In boys, both periodontal disease (RR, 1.69; 95% CI, 1.21-2.36) and caries (RR, 1.46; 95% CI, 1.04-2.05) were statistically significantly associated with the IMT. Adding teeth-brushing frequency to the model did not lead to a statistically significant association with the IMT or change the main result. The cumulative exposure to the risk factors during the follow-up time produced an RR of 1.03 (95% CI, 1.01-1.04; *P* = .002). When RRs in the 4 signs of oral infections category were considered, the E value for the whole population was 3.31 and for the boys was 3.93.

**Table 3.** Association of Oral Infections With Carotid Artery Intima-Media Thickness in 2007

Sign of Oral Infection	Dependent Variable: Third Tertile of Mean IMT in 2007, RR (95% CI)														
	All Participants					Boys					Girls				
	Age- and Sex-Adjusted	*P* Value	Multivariate^a^	*P* Value	Age-Adjusted		*P* Value	Multivariate^a^	*P* Value	Age-Adjusted		*P* Value	Multivariate^a^	*P* Value
Bleeding on probing and periodontal pockets														
Either	1.19 (0.96-1.48)	.12	1.21 (0.97-1.50)	.09	1.65 (1.19-2.29)		.003	1.69 (1.21-2.36)	.002	0.95 (0.70-1.28)		.73	0.96 (0.71-1.30)	.79
Both	1.23 (0.97-1.46)	.08	1.25 (0.99-1.59)	.06	1.81 (1.03-2.10)		.001	1.81 (1.28-2.57)	.001	0.90 (0.63-1.27)		.54	0.93 (0.65-1.31)	.67
Caries and fillings														
Either	1.50 (1.13-1.97)	.004	1.42 (1.07-1.88)	.01	1.54 (1.11-2.15)		.01	1.46 (1.04-2.05)	.02	1.40 (0.90-2.18)		.14	1.25 (0.79-1.97)	.35
Both	1.54 (1.16-2.03)	.003	1.46 (1.10-1.94)	.009	1.56 (1.11-2.17)		.01	1.49 (1.05-2.63)	.02	1.47 (0.94-2.30)		.09	1.30 (0.82-2.08)	.27
No. of signs of oral infections														
0	1 [Reference]		1 [Reference]		1 [Reference]			1 [Reference]		1 [Reference]			1 [Reference]	
1-3	1.82 (1.22-2.73)	.004	1.74 (1.16-2.62)	.008	1.84 (1.08-3.13)		.02	1.75 (1.02-3.00)	.04	1.73 (0.96-3.14)		.06	1.59 (0.86-2.91)	.14
4	2.04 (1.35-3.09)	.001	1.95 (1.28-3.00)	.002	2.43 (1.42-4.15)		.001	2.25 (1.30-3.89)	.004	1.61 (0.86-3.00)		.14	1.51 (0.80-2.85)	.20

Abbreviations: IMT, intima-media thickness; RR, risk ratio.

^a^ In addition to age and sex, the multivariate models were adjusted for cumulative exposure to the risk factors (1980-2007) and covariates were collected in 2007 (ie, family income and smoking). The results were corrected for selection bias owing to missing data by using the inverse probability weighting. Total participants: 489 (n = 218 boys; n = 271 girls).

Similar analyses for the 2001 IMT measurements, including the Poisson regression models, are presented in eTable 3 in the Supplement, the risk factors are shown in eTable 4 in the Supplement, and the linear regression analysis are shown in eTable 5 in the Supplement. In the linear regression, signs of caries (β, 0.199; *P* < .001) or any oral infections (β, 0.166; *P* = .004) were associated with the IMT. Overall, the trends were similar to those found in the 2007 follow-up.

## Discussion

By studying this unique population of the Cardiovascular Risk in Young Finns Study, we found that oral infections in childhood were associated with the development of subclinical carotid atherosclerosis 27 years later. The participants, all of whom were examined for signs of oral infections, including bleeding on probing, increased probing pocket depth, caries, or fillings, had an almost 2-fold risk for an increased IMT in their early middle age. An association between childhood oral infections with CVD risk factors, particularly high blood pressure and BMI, was also evident. However, the oral infections remained an independent risk factor of IMT after adjustment for a lifetime cumulative exposure to risk factors, including 31 separate measurements. The results show for the first time, to our knowledge, that childhood oral infections may be a modifiable risk factor for adult cardiovascular disease.

The role of childhood inflammatory diseases in atherogenesis has been reported in previous studies. Episodes of acute infections have been associated with carotid artery IMT in children,^17^ whereas conflicting results on their association with the adulthood risk of cardiovascular events have been reported.^18,19^ In the Cardiovascular Risk in Young Finns Study population, childhood infection–related hospitalizations have been associated significantly with a worse cardiovascular risk factor profile and atherosclerotic phenotype in adulthood.^20,21^ In addition, persistent *Chlamydia pneumoniae* infection may play a role in early lesion development.^22^ However, studies including examinations of oral infections in children are rare: pathological periodontal pockets were associated with diastolic blood pressure in obese adolescents,^23^ and accumulation of CVD risk factors was observed in 15-year-olds with caries compared with those without.^12^ To our knowledge, no long-term study data have been reported, and the results of the present study show that the cumulative exposure to both childhood and adulthood risk factors increased with the increasing number of oral infections.

Oral infections can contribute directly to atherosclerosis. They result from dysbiosis in the oral cavity, leading to host defense, including the clinical symptoms in susceptible individuals. The dysbiosis itself may have a role in systemic inflammation and insulin resistance.^24^ It possibly reflects a more widely distributed suboptimal microbiome^25^ that is present, for example, in type 2 diabetes.^26^ Progression of IMT has been shown to diminish with the improvement of clinical and microbiological periodontal status over a 3-year follow-up.^27^ Treatment of periodontitis (ie, intervening the subgingival microbiota) leads to improvements in inflammatory, thrombotic, and lipid biomarkers.^6^ Regardless of the mediators between oral dysbiosis and systemic inflammation, the outcome is similar: a phenotype with increased cardiometabolic risk factors, which were also observed in the present study.

It is possible that oral infections are biomarkers of poor oral hygiene. In the present study, 5.6% of girls and 12.2% of boys were reported not to brush their teeth daily. In Finland, all children and students are entitled to free oral health care, and adults have participated in communal dental care since 2001. Therefore, accessibility to dental treatment in Finland is not dependent on the family income. In the present study, frequency of teeth brushing was not associated with the socioeconomic status of the family. Therefore, neglecting daily oral hygiene routines may be accompanied by generally unhealthy behavior. Childhood socioeconomic status is associated with lifestyle factors and infectious burden in adulthood.^28,29^

Both in cross-sectional and long-term studies, boys were found to have thicker arterial walls and more adverse changes in vascular health than the age-matched girls.^30,31^ In the present study, the risk of subclinical atherosclerosis associated with oral infections was especially substantial in boys, although only the number of sites with increased periodontal probing depth and the brushing frequencies differed by sex. Male participants with all 4 signs of oral infections in childhood had a 125% increased risk for high IMT values 27 years later. It is known from this and other cohorts that the association of childhood risk factors may be stronger in males than females and that, overall, being male is a risk factor for atherosclerosis, which may lead to CVD events approximately a decade sooner for men than women.^8^ Furthermore, destructive periodontal disease is more prevalent in men,^32^ probably owing not only to behavioral and environmental dissimilarities but also to sex-based differences in immunologic and inflammatory responses.

### Limitations

The limitations of this study include the small number of participants with IMT measurements and the slight difference in the number of girls and boys examined. However, we corrected the models for a putative selection bias by weighting with an inverse probability score, which resulted in smaller CIs of the RRs and estimates. In addition, the large E values for the main results increase credibility, although we cannot rule out unmeasured confounding. The clinical oral examination was based on data registered only from the index teeth. It was not feasible to repeat the oral examinations in 2007, which would have been of interest in relation to childhood factors of adulthood oral health status. We had neither information on the dental treatments during the follow-up nor the data on nutrient intake and diet. Plaque or saliva samples were not collected during the examination, and thus, microbiological profiling was not possible.

## Conclusions

This study, in which participants were followed up for 27 years, suggests that oral infections in childhood are associated with the subclinical carotid atherosclerosis in adulthood.

## Notes

Supplement. **eTable 1.** Cutoff Values for the Classification of the Risk Factors **eTable 2.** Baseline Comparisons of Subjects With and Without IMT Measurements in 2007 **eTable 3.** Association of Continuous Oral Infection Parameters With IMT 2001 and 2007 **eTable 4.** Characteristics of the Population in 2001 and 2007 According to Baseline Age Groups in 1980 **eTable 5.** Linear Regression Analysis for the Association of IMT in 2001 With the Signs of Oral Infections **eFigure.** Distribution of Oral Health Parameters Registered in Clinical Examination 1980

## References

[1] Suominen AL, Varsio S, Helminen S, Nordblad A, Lahti S, Knuuttila M Dental and periodontal health in Finnish adults in 2000 and 2011. Acta Odontol Scand. 2018;76(5):-. doi:10.1080/00016357.2018.1451653 29546776

[2] Kämppi A, Tanner T, Päkkilä J, Geographical distribution of dental caries prevalence and associated factors in young adults in Finland. Caries Res. 2013;47(4):346-354. doi:10.1159/000346435 23548873

[3] Bimstein E, Huja PE, Ebersole JL The potential lifespan impact of gingivitis and periodontitis in children. J Clin Pediatr Dent. 2013;38(2):95-99. doi:10.17796/jcpd.38.2.j525742137780336 24683769

[4] Lockhart PB, Bolger AF, Papapanou PN, ; American Heart Association Rheumatic Fever, Endocarditis, and Kawasaki Disease Committee of the Council on Cardiovascular Disease in the Young, Council on Epidemiology and Prevention, Council on Peripheral Vascular Disease, and Council on Clinical Cardiology Periodontal disease and atherosclerotic vascular disease: does the evidence support an independent association? a scientific statement from the American Heart Association. Circulation. 2012;125(20):2520-2544. doi:10.1161/CIR.0b013e31825719f3 22514251

[5] Pietiäinen M, Liljestrand JM, Kopra E, Pussinen PJ Mediators between oral dysbiosis and cardiovascular diseases. Eur J Oral Sci. 2018;126(suppl 1):26-36. doi:10.1111/eos.12423 30178551

[6] Teeuw WJ, Slot DE, Susanto H, Treatment of periodontitis improves the atherosclerotic profile: a systematic review and meta-analysis. J Clin Periodontol. 2014;41(1):70-79. doi:10.1111/jcpe.12171 24111886

[7] Liljestrand JM, Mäntylä P, Paju S, Association of endodontic lesions with coronary artery disease. J Dent Res. 2016;95(12):1358-1365. doi:10.1177/0022034516660509 27466397

[8] Raitakari OT, Juonala M, Kähönen M, Cardiovascular risk factors in childhood and carotid artery intima-media thickness in adulthood: the Cardiovascular Risk in Young Finns Study. JAMA. 2003;290(17):2277-2283. doi:10.1001/jama.290.17.2277 14600186

[9] Laitinen TT, Pahkala K, Magnussen CG, Lifetime measures of ideal cardiovascular health and their association with subclinical atherosclerosis: the Cardiovascular Risk in Young Finns Study. Int J Cardiol. 2015;185:186-191. doi:10.1016/j.ijcard.2015.03.051 25797675

[10] Merchant AT, Nahhas GJ, Wadwa RP, Periodontal microorganisms and cardiovascular risk markers in youth with type 1 diabetes and without diabetes. J Periodontol. 2016;87(4):376-384. doi:10.1902/jop.2015.150531 26616842

[11] Lempert SM, Froberg K, Christensen LB, Kristensen PL, Heitmann BL Association between body mass index and caries among children and adolescents. Community Dent Oral Epidemiol. 2014;42(1):53-60. doi:10.1111/cdoe.12055 23763718

[12] Larsson B, Johansson I, Hallmans G, Ericson T Relationship between dental caries and risk factors for atherosclerosis in Swedish adolescents? Community Dent Oral Epidemiol. 1995;23(4):205-210. doi:10.1111/j.1600-0528.1995.tb00232.x 7587140

[13] World Medical Association World Medical Association Declaration of Helsinki: ethical principles for medical research involving human subjects. JAMA. 2013;310(20):2191-2194. doi:10.1001/jama.2013.281053 24141714

[14] Raitakari OT, Juonala M, Rönnemaa T, Cohort profile: the Cardiovascular Risk in Young Finns Study. Int J Epidemiol. 2008;37(6):1220-1226. doi:10.1093/ije/dym225 18263651

[15] Rovio SP, Pahkala K, Nevalainen J, Cardiovascular risk factors from childhood and midlife cognitive performance: the Young Finns Study. J Am Coll Cardiol. 2017;69(18):2279-2289. doi:10.1016/j.jacc.2017.02.060 28473132

[16] VanderWeele TJ, Ding P Sensitivity analysis in observational research: introducing the E-value. Ann Intern Med. 2017;167(4):268-274. doi:10.7326/M16-2607 28693043

[17] Liuba P, Persson J, Luoma J, Ylä-Herttuala S, Pesonen E Acute infections in children are accompanied by oxidative modification of LDL and decrease of HDL cholesterol, and are followed by thickening of carotid intima-media. Eur Heart J. 2003;24(6):515-521. doi:10.1016/S0195-668X(02)00750-9 12643884

[18] Pesonen E, Andsberg E, Ohlin H, Dual role of infections as risk factors for coronary heart disease. Atherosclerosis. 2007;192(2):370-375. doi:10.1016/j.atherosclerosis.2006.05.018 16780845

[19] Qanitha A, de Mol BA, Pabittei DR, Infections in early life and premature acute coronary syndrome: a case-control study. Eur J Prev Cardiol. 2016 t;23(15):1640-1648. doi:10.1177/2047487316640656 27006417

[20] Liu RS, Burgner DP, Sabin MA, Childhood infections, socioeconomic status, and adult cardiometabolic risk. Pediatrics. 2016;137(6):e20160236. doi:10.1542/peds.2016-0236 27235447

[21] Burgner DP, Sabin MA, Magnussen CG, Early childhood hospitalisation with infection and subclinical atherosclerosis in adulthood: the Cardiovascular Risk in Young Finns Study. Atherosclerosis. 2015;239(2):496-502. doi:10.1016/j.atherosclerosis.2015.02.024 25721701

[22] Volanen I, Järvisalo MJ, Vainionpää R, Increased aortic intima-media thickness in 11-year-old healthy children with persistent *Chlamydia pneumoniae* seropositivity. Arterioscler Thromb Vasc Biol. 2006;26(3):649-655. doi:10.1161/01.ATV.0000202664.76816.bb 16397138

[23] Zeigler CC, Wondimu B, Marcus C, Modéer T Pathological periodontal pockets are associated with raised diastolic blood pressure in obese adolescents. BMC Oral Health. 2015;15:41. doi:10.1186/s12903-015-0026-6 25884594 PMC4373518

[24] Demmer RT, Breskin A, Rosenbaum M, The subgingival microbiome, systemic inflammation and insulin resistance: the Oral Infections, Glucose Intolerance and Insulin Resistance Study. J Clin Periodontol. 2017;44(3):255-265. doi:10.1111/jcpe.12664 27978598 PMC5328907

[25] Nakajima M, Arimatsu K, Kato T, Oral administration of *P. gingivalis* induces dysbiosis of gut microbiota and impaired barrier function leading to dissemination of enterobacteria to the liver. PLoS One. 2015;10(7):e0134234. doi:10.1371/journal.pone.0134234 26218067 PMC4517782

[26] Wang J, Jia H Metagenome-wide association studies: fine-mining the microbiome. Nat Rev Microbiol. 2016;14(8):508-522. doi:10.1038/nrmicro.2016.83 27396567

[27] Desvarieux M, Demmer RT, Jacobs DR, Papapanou PN, Sacco RL, Rundek T Changes in clinical and microbiological periodontal profiles relate to progression of carotid intima-media thickness: the Oral Infections and Vascular Disease Epidemiology study. J Am Heart Assoc. 2013;2(6):e000254. doi:10.1161/JAHA.113.000254 24166489 PMC3886779

[28] Puolakka E, Pahkala K, Laitinen TT, Childhood socioeconomic status and lifetime health behaviors: the Young Finns Study. Int J Cardiol. 2018;258:289-294. doi:10.1016/j.ijcard.2018.01.088 29428239

[29] Palm F, Pussinen PJ, Aigner A, Association between infectious burden, socioeconomic status, and ischemic stroke. Atherosclerosis. 2016;254:117-123. doi:10.1016/j.atherosclerosis.2016.10.008 27728851

[30] Osika W, Dangardt F, Montgomery SM, Volkmann R, Gan LM, Friberg P Sex differences in peripheral artery intima, media and intima media thickness in children and adolescents. Atherosclerosis. 2009;203(1):172-177. doi:10.1016/j.atherosclerosis.2008.05.054 18603254

[31] Chen Y, Dangardt F, Osika W, Berggren K, Gronowitz E, Friberg P Age- and sex-related differences in vascular function and vascular response to mental stress: longitudinal and cross-sectional studies in a cohort of healthy children and adolescents. Atherosclerosis. 2012;220(1):269-274. doi:10.1016/j.atherosclerosis.2011.10.030 22078247

[32] Page RC, Beck JD Risk assessment for periodontal diseases. Int Dent J. 1997;47(2):61-87. doi:10.1111/j.1875-595X.1997.tb00680.x 9448791

