# Supplementary material for: Association of Childhood Oral Infections With Cardiovascular Risk Factors and Subclinical Atherosclerosis in Adulthood
Source: JAMA Netw Open. 2019 Apr 26;2(4):e192523. doi: 10.1001/jamanetworkopen.2019.2523 (PMC6487573; doi:10.1001/jamanetworkopen.2019.2523)
Supplement: Supplement. — eTable 1. Cutoff Values for the Classification of the Risk Factors eTable 2. Baseline Comparisons of Subjects With and Without IMT Measurements in 2007 eTable 3. Association of Continuous Oral Infection Parameters With IMT 2001 and 2007 eTable 4. Characteristics of the Population in 2001 and 2007 According to Baseline Age Groups in 1980 eTable 5. Linear Regression Analysis for the Association of IMT in 2001 With the Signs of Oral Infections eFigure. Distribution of Oral Health Parameters Registered in Clinical Examination 1980 [file jamanetwopen-2-e192523-s001.pdf]

Supplementary Online Content

Pussinen PJ, Paju S, Koponen J, et al. Association of childhood oral infections with cardiovascular risk factors and subclinical atherosclerosis in adulthood. *JAMA Netw Open.* 2019;2(4): e192523. doi:10.1001/jamanetworkopen.2019.2523

eTable 1. Cutoff Values for the Classification of the Risk Factors

eTable 2. Baseline Comparisons of Subjects With and Without IMT Measurements in 2007

eTable 3. Association of Continuous Oral Infection Parameters With IMT 2001 and 2007

eTable 4. Characteristics of the Population in 2001 and 2007 According to Baseline Age Groups in 1980

eTable 5. Linear Regression Analysis for the Association of IMT in 2001 With the Signs of Oral Infections

eFigure. Distribution of Oral Health Parameters Registered in Clinical Examination 1980

This supplementary material has been provided by the authors to give readers additional information about their work.

©2019 Pussinen PJ et al. *JAMA Network Open.*

eTable 1. Cutoff Values for the Classification of the Risk Factors

Risk factor	Year	Cut off value	Unit
Systolic blood pressure	1980	113.3	mmHg
	1983	115.6	
	1986	118.5	
	2001	117.5	
	2007	121.5	
Diastolic blood pressure	1980	73.6	mmHg
	1983	70.5	
	1986	71.5	
	2001	74.5	
	2007	78.5	
LDL cholesterol	1980	156.4	mg/dl
	1983	137.7	
	1986	121.8	
	2001	129.5	
	2007	128.6	
HDL cholesterol	1980	69.2	mg/dl
	1983	72.3	
	1986	63.0	
	2001	61.1	
	2007	62.8	
Triglycerides	1980	60.0	mg/dl
	1983	82.3	
	1986	92.2	
	2001	128.4	
	2007	124.9	

©2019 Pussinen PJ et al. *JAMA Network Open.*

Plasma glucose	1986	86.4	mmol/l
	2001	93.1	
	2007	97.2	
Body mass index	1980	17.12	kg/m^2
	1983	19.59	
	1986	21.62	
	2001	25.76	
	2007	27.40	
CRP	1980	0.456	mg/l
	2001	1.845	
	2007	1.540	

The thresholds are determined by using the ROC analysis. The state variable was mean IMT in 2007, test variables were the risk factors one by one. The cut-off values presented in this table were selected from the area-under-curve displaying a specificity of 0.75.

©2019 Pussinen PJ et al. *JAMA Network Open.*

eTable 2. Baseline Comparisons of Subjects With and Without IMT Measurements in 2007

**Variable**		**With IMT**	**Without IMT**	**p-value**
		**N (%)[a]**		
N		489 (64.8)	266 (35.2)	
Males		218 (44.6)	153 (57.5)	0.001
Signs of periodontal disease		383 (81.5)	214 (83.6)	0.48
	Bleeding on probing	311 (63.6)	181 (68.0)	0.22
	Periodontal pockets	258 (54.7)	132 (51.6)	0.42
Signs of caries		418 (85.5)	238 (89.5)	0.12
	Fillings	418 (85.5)	238 (89.5)	0.12
	Caries	402 (82.2)	219 (82.3)	0.97
Signs of oral infections	0	26 (5.5)	7 (2.7)	0.33
	1	27 (5.7)	14 (5.5)	
	2	81 (17.2)	46 (18.0)	
	3	172 (36.4)	107 (41.8)	
	4	166 (35.2)	82 (32.0)	
		**Mean (SD)[b]**		
Age (years)		7.99 (2.05)	8.10 (1.93)	0.48
BMI (kg/m^2)		16.8 (2.2)	16.7 (9.5)	0.91
Systolic blood pressure (mmHg)		110 (9.7)	110 (9.7)	0.50
Diastolic blood pressure (mmHg)		67.9 (9.3)	67.5 (9.2)	0.56
Total cholesterol (mg/dl)		209.2 (34.0)	205.3 (34.4)	0.15
LDL cholesterol (mg/dl)		135.3 (30.9)	131.1 (31.3)	0.08
HDL cholesterol (mg/dl)		63.0 (12.0)	63.4 (12.4)	0.64
Triglycerides (mg/dl)		53.1 (27.5)	53.1 (23.0)	0.93
CRP (mg/l)[c, d]		0.83 (2.59)	0.90 (1.87)	0.53
Family income (among 8 classes)		5.36 (1.74)	5.14 (1.83)	0.11
Percentage of sites bleeding on probing[c]		20.2 (21.0)	21.4 (20.7)	0.21
Percentage of sites with periodontal pockets[c]		14.1 (18.4)	13.8 (18.1)	0.56
Number of teeth with fillings[c]		4.5 (3.4)	4.7 (3.6)	0.65
Number of surfaces with caries[c]		9.3 (7.8)	9.4 (7.5)	0.70

©2019 Pussinen PJ et al. *JAMA Network Open.*

[a] Chi-square test; [b] t-test; [c] t-test after log-transformation; [d] n=76 without IMT, n=410 with IMT

©2019 Pussinen PJ et al. *JAMA Network Open.*

eTable 3. Association of Continuous Oral Infection Parameters With IMT 2001 and 2007

	Dependent: 3rd tertile of mean IMT in 2001		Dependent: 3rd tertile of mean IMT in 2007	
	**RR (95% CI), p-value**			
**Sign of oral infection**	**Age-and sex-adjusted**	**Multivariate[a]**	**Age-and sex-adjusted**	**Multivariate[a]**
Number of sites with bleeding on probing	0.975 (0.883-1.077), 0.62	0.959 (0.869-1.057), 0.40	1.015 (0.991-1.040), 0.23	1.021 (0.978-1.065), 0.35
Number of sites with increased periodontal pocket depth	1.015 (0.962-1.072), 0.58	1.017 (0.964-1.072), 0.54	1.013 (0.999-1.007), 0.19	1.014 (0.989-1.039), 0.27
Number of teeth with fillings	1.076 (1.016-1.139), 0.01	1.063 (1.005-1.125), 0.03	1.023 (0.996-1.050), 0.09	1.024 (0.997-1.053), 0.08
Number of surfaces with caries	1.025 (1.001-1.051), 0.04	1.074 (0.996-1.046), 0.09	1.010 (0.999-1.021), 0.08	1.011 (0.999-1.022), 0.08
All signs of oral infections[b]	1.057 (0.992-1.127), 0.08	1.046 (0.981-1.116), 0.17	1.032 (1.001-1.063), 0.04	1.031 (1.000-1.064), 0.04
Any sign of oral infection[c]	1.895 (0.920-3.905), 0.08	1.791 (0.869-3.692), 0.11	1.868 (1.249-2.795), 0.002	1.777 (1.182-2.670), 0.006

[a] In addition to age and sex adjusted for covariates: family income, smoking, and cumulative exposure to the risk factors, and inverse probability weighting. The models for mean IMT in 2001 were adjusted for the covariates collected until or in 2001, the models for mean IMT in 2007 were adjusted for covariates collected until or in 2007. The results are corrected for selection bias due to missing data by using the inverse probability weighting. All n=468 (2001), n=489 (2007).

[b] Bleeding on probing, probing pocket depth, fillings, and caries; sum of standardized values.

[c] Presence of at least one of the 4 signs of oral infection: bleeding on probing, periodontal probing pocket depth, fillings, or caries.

eTable 4. Characteristics of the Population in 2001 and 2007 According to Baseline Age Groups in 1980

Examination year	All	Age 6 years	Age 9 years	Age 12 years
	**Mean (SD)**			
**Examination in 2001**				
Age (years)	29.0 (2.0)	26.3 (0.27)	28.8 (0.27)	31.3 (0.26)
Mean IMT (mm)	0.576 (0.084)	0.557 (0.069)	0.583 (0.091)	0.583 (0.086)
BMI (kg/m^2)	25.0 (4.1)	24.3 (4.2)	25.7 (4.4)	24.9 (3.6)
Systolic blood pressure (mmHg)	115 (14)	112 (13)	117 (15)	115 (14)
Diastolic blood pressure (mmHg)	70.3 (10.3)	67.5 (9.4)	71.0 (10.8)	71.6 (11.0)
Total cholesterol (mg/dl)	196.4 (34.8)	192.6 (36.4)	196.1 (31.3)	198.4 (36.7)
LDL cholesterol (mg/dl)	123.4 (29.8)	118.7 (30.9)	124.9 (26.3)	125.3 (31.7)
HDL cholesterol (mg/dl)	49.9 (12.0)	50.7 (12.4)	47.9 (11.6)	50.7 (12.0)
Triglycerides (mg/dl)	118.7 (66.4)	116.9 (67.3)	116.9 (58.5)	122.2 (73.5)
Glucose (mg/dl)	90.4 (16.6)	89.3 (9.9)	91.1 (16.4)	90.5 (20.5)
	**Median (IQR)**			
CRP (mg/l)	0.99 (1.86)	1.29 (2.17)	0.86 (1.65)	0.91 (1.73)
**Examination in 2007**				
Age (years)	35.0 (2.0)	32.3 (0.27)	34.8 (0.27)	37.3 (0.26)
Mean IMT (mm)	0.615 (0.080)	0.592 (0.067)	0.619 (0.086)	0.631 (0.079)
BMI (kg/m^2)	26.0 (4.6)	25.2 (4.2)	26.6 (5.2)	26.2 (4.2)
Systolic blood pressure (mmHg)	119 (13)	116 (12)	120 (14)	121 (14)
Diastolic blood pressure (mmHg)	75.2 (10.8)	72.7 (10.4)	75.9 (10.5)	76.7 (11.2)
Total cholesterol (mg/dl)	192.2 (33.3)	187.2 (30.9)	194.5 (33.6)	194.5 (34.8)
LDL cholesterol (mg/dl)	116.8 (28.6)	113.3 (25.9)	118.7 (27.8)	117.9 (30.6)
HDL cholesterol (mg/dl)	51.8 (12.4)	51.8 (12.8)	50.7 (12.8)	51.4 (12.0)
Triglycerides (mg/dl)	123.1 (78.0)	110.7 (66.4)	126.7 (65.5)	127.6 (95.7)
Glucose (mg/dl)	95.8 (16.9)	92.9 (7.2)	97.2 (21.2)	96.8 (17.6)
	**Median (IQR)**			
CRP (mg/l)	0.96 (1.97)	0.96 (1.81)	1.23 (2.76)	0.88 (1.74)

©2019 Pussinen PJ et al. *JAMA Network Open.*

eTable 5. Linear Regression Analysis for the Association of IMT in 2001 With the Signs of Oral Infections

**Independent variables**	**Dependent: mean IMT in 2001**		
	**Beta (SE)**	**p-value**	**R^2**
Signs of periodontal disease [a]	0.088	0.12	0.009
Signs of caries [b]	0.199	<0.001	0.029
Signs of oral infections [c]	0.166	0.004	0.031

Multiple linear regression adjusted for the cumulative exposure to CVD risk factors during the follow-up classified by using the AUC-method. The models are weighed by using inverse probability score.

R^2 values are reported from corresponding unadjusted simple linear regressions with fitting models. Statistically significant results are highlighted in bold.

[a] Number of sites with signs of periodontal disease (bleeding on probing and increased probing pocket depth)

[b] Sum of standardized values for number of surfaces with caries and teeth with fillings.

[c] Number of signs of oral infections (bleeding on probing, increased probing pocket depth, caries, and fillings).

©2019 Pussinen PJ et al. *JAMA Network Open.*

eFigure. Distribution of Oral Health Parameters Registered in Clinical Examination 1980

A) Number of surfaces with caries; B) number of teeth with fillings; C) percentage of examined sites with bleeding on probing; and D) percentage of examined sites with increased periodontal probing depth.

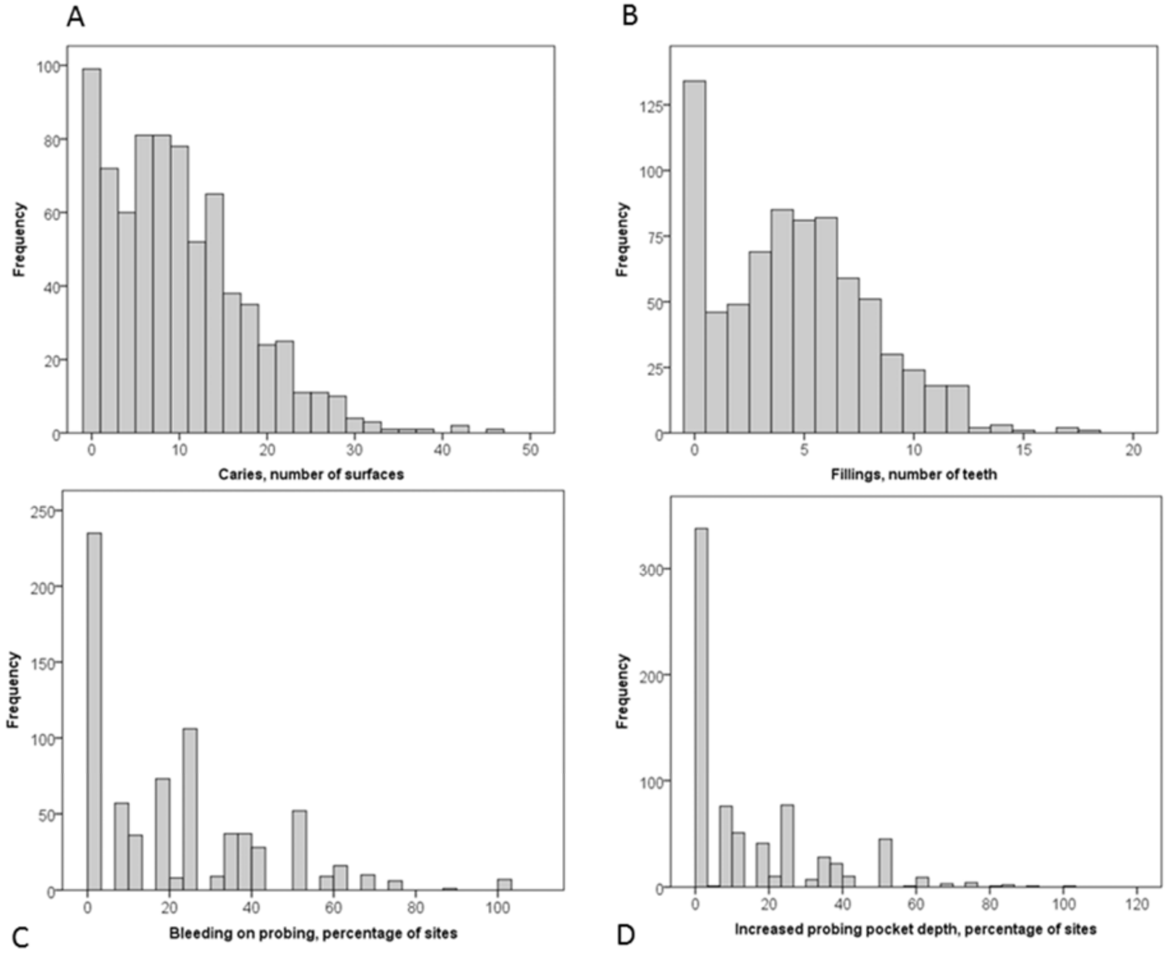

©2019 Pussinen PJ et al. *JAMA Network Open.*
